# Characterization and wearability evaluation of a fully portable wrist exoskeleton for unsupervised training after stroke

**DOI:** 10.1186/s12984-020-00749-4

**Published:** 2020-10-07

**Authors:** Charles Lambelet, Damir Temiraliuly, Marc Siegenthaler, Marc Wirth, Daniel G. Woolley, Olivier Lambercy, Roger Gassert, Nicole Wenderoth

**Affiliations:** 1 Neural Control of Movement Lab, Department of Health Sciences and Technology, ETH Zurich, Zurich, Switzerland; 2 Rehabilitation Engineering Laboratory, Department of Health Sciences and Technology, ETH Zurich, Zurich, Switzerland

**Keywords:** Wearable exoskeleton, Wrist, Stroke, Robotic rehabilitation, Home-based, Wearability/usability evaluation, Donning/doffing, Unsupervised training, Admittance control, eWrist

## Abstract

**Background:**

Chronic hand and wrist impairment are frequently present following stroke and severely limit independence in everyday life. The wrist orientates and stabilizes the hand before and during grasping, and is therefore of critical importance in activities of daily living (ADL). To improve rehabilitation outcomes, classical therapy could be supplemented by novel therapies that can be applied in unsupervised settings. This would enable more distributed practice and could potentially increase overall training dose. Robotic technology offers new possibilities to address this challenge, but it is critical that devices for independent training are easy and appealing to use. Here, we present the development, characterization and wearability evaluation of a fully portable exoskeleton for active wrist extension/flexion support in stroke rehabilitation.

**Methods:**

First we defined the requirements, and based on these, constructed the exoskeleton. We then characterized the device with standardized haptic and human-robot interaction metrics. The exoskeleton is composed of two modules placed on the forearm/hand and the upper arm. These modules weigh 238 g and 224 g, respectively. The forearm module actively supports wrist extension and flexion with a torque up to 3.7 Nm and an angular velocity up to 530 deg/s over a range of 154^∘^. The upper arm module includes the control electronics and battery, which can power the device for about 125 min in normal use. Special emphasis was put on independent donning and doffing of the device, which was tested via a wearability evaluation in 15 healthy participants and 2 stroke survivors using both qualitative and quantitative methods.

**Results:**

All participants were able to independently don and doff the device after only 4 practice trials. For healthy participants the donning and doffing process took 61 ±15 s and 24 ±6 s, respectively. The two stroke survivors donned and doffed the exoskeleton in 54 s/22 s and 113 s/32 s, respectively. Usability questionnaires revealed that despite minor difficulties, all participants were positive regarding the device.

**Conclusions:**

This study describes an actuated wrist exoskeleton which weighs less than 500 g, and which is easy and fast to don and doff with one hand. Our design has put special emphasis on the donning aspect of robotic devices which constitutes the first barrier a user will face in unsupervised settings. The proposed device is a first and intermediate step towards wearable rehabilitation technologies that can be used independently by the patient and in unsupervised settings.

## Background

Stroke affects approximately 795’000 people each year in the US alone and is one of the leading causes of long-term adult disability and dependency [1]. Traditional stroke rehabilitation options for outpatients include therapist-based treatments with hands-on physical and occupational therapy in rehabilitation centres. The treatment lasts several weeks and is composed of periodic blocked practice, but overall training time remains low compared to the time the patient is inactive at home [2, 3]. Moreover, stroke patients are discharged at an increasingly early stage [4, 5] requiring new approaches for rehabilitation training in unsupervised settings. These novel approaches must be effective [6, 7], and empower patients to self-initiate rehabilitation training that will enable more distributed sessions. This is particularly important since, in the future, more rehabilitation resources will be moved to community settings and patient homes to complement conventional therapy [8–11].

Upper extremity hemiparesis is a common weakness following stroke and heavily impairs ADL [12]. Adequate wrist function is critical for orientating and stabilising the hand [13], but the recovery process of this specific joint is still not well understood in stroke survivors [14]. It has been shown that the probability of recovering distal functions (e.g. the wrist) are closely linked with the acute state of proximal functions (shoulder or elbow) [15]. In the same vein, distal training can lead to positive effects at the shoulder and elbow [16–18]. While the hand has received a lot of attention from the research community, there remains a need to provide wrist function training.

Robot-assisted therapy for stroke patients is a promising approach [19, 20] and proven advantages include: 1) increasing dose and intensity of training [21–23], 2) allowing quantitative measurements to assess performance and recovery of the patient more precisely than conventional rehabilitation training [24], and 3) engaging the patient in a motivating and stimulating environment [25, 26]. However, a robot-mediated therapy administered in unsupervised settings implies several technical, clinical and social challenges: first of all, the technology must be safe to be deployed in such a context, its footprint acceptable to the patient, relatives and caregivers, and it should adhere to conventional therapy principles to administer appropriate treatment to the user. Moreover, the device must be adaptable to the individual and designed such that patients can use it independently and in various environmental settings [27–29].

A myriad of devices have targeted training of the whole arm, and also more specifically the hand and fingers [19, 20, 30], while relatively few wearable exoskeletons have focused on the wrist [31–35]. Unlike stationary rehabilitation devices [36–38], a fully wearable exoskeleton offers the possibility to use (i.e. to train) the paretic limb during functional everyday tasks [7, 39, 40] where higher training dose could more conveniently be achieved. Exoskeletons interact at the level of individual joints and enable joint specific kinematic assessments [41, 42]. Moreover, it has been shown that training isolated individual joint movements facilitates learning complex multi-joint movements [43, 44]. Practically, this means that through the *“part-whole transfer paradigm”* simple low degree of freedom (DoF) robotic devices could facilitate the training of more complex movements. In an unsupervised training context, simplicity is paramount [45], therefore, simple wearable technologies might provide an interesting add-on to a conventional therapy where complex movements are trained.

We have previously presented a first prototype version of the *eWrist* [46]. Here we present further developments which focussed on improving portability, independence of use and adaptability in view of unsupervised use of the system. The *eWrist* is a fully wearable single DoF sEMG-based force controlled wrist exoskeleton that actively supports extension and flexion. We put special emphasis on the attachment mechanisms that facilitate the donning and doffing of the device so that a hemiparetic patient could mount the device independently with a single hand. Among the vast amount of published work on rehabilitation devices for in-home therapy, few have addressed the fixation issue, which constitutes the first barrier a user would have to overcome in order to use the device independently [47, 48]. Currently the *eWrist* is intended to be used as a training device rather than as an assistive exoskeleton during ADL. However, our long term design goal is to fuse training and assistance with the aim of increasing movement of the affected arm in daily life via technology that modulates assistance in order to improve upper arm function. This requires an exoskeleton that is fully wearable, easy to use, and especially simple to don and doff. The *eWrist* is our first wearable prototype that is capable of assisting wrist flexion and extension, the latter being particularly relevant for post stroke recovery [49].

Here we briefly describe the previous *eWrist* version, we then outline requirements for a fully wearable wrist exoskeleton and present an advanced *eWrist* device where we focussed on wearability improvements. We first characterize the current implementation based on standardized haptic and human-robot interaction metrics for rehabilitation devices. Secondly, we present the results of a wearability study which evaluates the donning/doffing procedure in healthy and stroke participants. Finally, limitations of the current work and potential future use of the *eWrist* are discussed.

## Methods

### Previous version of the eWrist

We previously introduced the *eWrist* [46], an exoskeleton actuated by a DC motor via bevel gears that actively supports wrist extension/flexion movements, measures force exerted on the handle, absolute angular position and velocity at the wrist axis via a Hall sensor integrated on the motor shaft. This prototype had several shortcomings, the major one being the overall weight of the exoskeleton (505 g total weight, of which 340 g was located on the forearm and hand). The current version of the *eWrist* includes the following improvements: (i) lowering the weight of the forearm module and reducing its physical profile by implementing a lighter and smaller motor, and by moving as many components as possible to more proximal areas, (ii) increasing the durability of the *eWrist* by implementing metal gears and an absolute angular Hall encoder, (iii) integrating an improved electronic design to simplify debugging and interaction with the device, and (iv) facilitating the overall donning/doffing via a completely redesigned mechanism for the upper arm module.

### Design requirements

Our aims were to reduce the distal weight of the *eWrist* and most importantly to develop user-friendly mechanisms that allow one-handed donning and doffing of the whole exoskeleton. In the following sections we establish the requirements.

#### Transmission type

Three general transmission types are commonly seen in wearable exoskeletons, namely: pneumatic, cable-driven and linear actuators (DC motors) [50]. Pneumatic systems are compliant and adapt their shape to the human body but accurate control is difficult to implement because of non-linearities. Moreover, several components such as pump, reservoir, regulator and valves are inherent to these systems which make the integration into fully wearable solutions tedious [33, 51, 52]. Cable-driven systems offer high compliance and low physical profile at the distal extremity while requiring less supplementary components then pneumatics. However, backlash and transmission losses make such systems challenging to control [53–55]. Linear actuators and direct DC motor actuation are straightforward to implement and allow high controllability of position, speed and torque. Nevertheless, special attention to weight and backdrivability must be paid when placed distally and directly mounted to the paretic limb [32, 56–58].

#### Actuation output torque, velocity and RoM

A minimal RoM of 140^∘^ (70^∘^ in flexion and extension) and an output torque at the wrist up to 3 Nm were chosen as design criteria based on previous work [13, 59, 60]. An angular velocity up to 180 deg/s (3.14 rad/s) was considered appropriate in a rehabilitation context, and subsequently in a daily life assistive context.

#### Sensing

When backdrivability of a transmission mechanism is not ensured, i.e. force (torque) cannot be assessed in the reverse direction (i.e. from limb to motor) by measuring the motor’s current draw, a common solution is to implement a force/torque sensor (load cell) serially connected with the joint kinematics [61]. Moreover, the absolute angular position of the wrist joint is needed and can either be achieved through initialization of motor encoders or with an additional absolute angular sensor.

#### Anatomical positioning

Compliant exoskeletons adapt to the biological joint and therefore do not require precise positioning [33, 62]. Rigid exoskeletons on the other hand, although much easier to control, need their mechanical axes to be aligned to the anatomical joint in order to not hinder movements or cause discomfort [32, 57, 63].

#### Fixation

Attachment systems play a major role in the ergonomics and usability of wearable devices [47, 48]. Velcro and straps are a common, quickly implemented and therefore favoured solution to attach exoskeletons to the human body [56, 64]. However, these fixation techniques can be highly challenging if the user has to perform them with a single hand. For that reason, novel techniques need to be implemented to ensure that the whole exoskeleton attachment can be performed with a single hand and in reasonable time (<2 min) [65, 66]. Furthermore, the fixation systems must fulfil certain requirements in term of attachment strength and stability, and should also remain compliant to changes in body shape during movements [67].

#### Weight, size and ergonomics

Stroke survivors are highly sensitive to mechanical loads applied on their paretic limb, and even more so when the load is located distally [68, 69]. Moreover, an acceptable weight for a wrist exoskeleton is subjective and essentially patient specific [70, 71]. According to a previous study [53], an ideal upper benchmark weight for a wrist exoskeleton placed distally is 250g. This is often achieved by moving parts that are not directly required for actuation (e.g. battery, controller and others) to more proximal body parts [39, 53, 72].

In order to limit the creation of shear forces and pressure on the skin, a short fixation structure is preferred for the forearm part of the exoskeleton. In this way, the pronation and supination of the forearm is less hindered and ergonomics enhanced [63, 73]. Regarding fixation to the hand, a palm free of attachments is desired to promote hand interaction with the environment [33, 53].

Finally, for the sake of ergonomics, the donning process and ultimately set-up time, the wearable device should be entirely located on the arm or in a position that does not hinder any movements of other joints (e.g. elbow/shoulder) or actions (e.g. sitting on a chair or lying on a bed) of the user [67].

### Design implementation

**Fig. 1:**
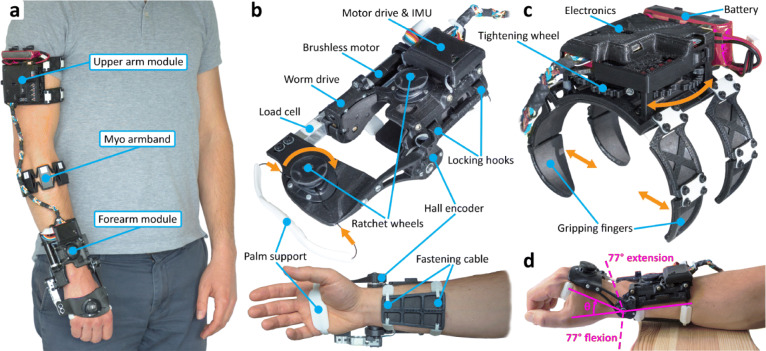
**a** The current version of the *eWrist* mounted on the right arm of a user. It is composed of three modules, namely, the forearm module, the upper arm module and the Myo armband. **b** The forearm module of the *eWrist* with two ratchet wheels (on the handle and on the forearm) to adjust the tightening tension. The locking hooks are equipped with two guiding magnets to ease the fixation. **c** The upper arm module of the *eWrist* and the four compliant gripping fingers that move inwards when the tightening wheel is turned. With the press of a button fixed to a ratchet (not shown on the picture) the tightening wheel unwinds and the fingers move outwards. **d** The restrained RoM from 77^∘^ in flexion to 77^∘^ in extension

Based on the requirements, the design of the *eWrist* focussed on lowering its physical profile and weight, enhancing wear comfort, and increasing usability of the fixation system for the exoskeleton, battery and electronics. To reduce weight on the distal part of the arm, the battery and electronics have been placed on the upper arm (upper arm module) while the actuated part of the exoskeleton is on the forearm and hand (forearm module) as depicted in Fig. 1a. Except for the motor, the motor drive, the worm drive and the Myo armband, all components are low-cost and widely available.

#### Structure and fixation

All structural parts of the *eWrist* are 3D printed in PLA^1^ which is a rigid and lightweight polyester. Parts requiring high flexibility are 3D printed in TPU^2^ which is a soft and elastic polymer allowing for more compliance around limbs and comfort on the skin. 3D printing techniques offer highly iterative design processes which facilitate the mechanical development, and allow adaptation of the *eWrist* to different user sizes.

*Forearm module:* The forearm module weighs 238 g and is attached to the forearm and hand (with the handle) (Fig. 1b). The design imposes 1 DoF at the wrist, which actively supports flexion/extension while preventing radial and ulnar wrist deviation. It allows a mechanical RoM in extension and flexion up to 103^∘^ and 112^∘^, respectively (i.e. 215^∘^ overall), but it has been limited to ±77^∘^ (i.e. restrained RoM = 154^∘^) based on the average wrist RoM [74]. Pronosupination of the forearm is not actively supported, but is also not hindered. The procedure to don the forearm module requires the user to (i) place the hand inside the loop formed by the palm support and position the device on the forearm and hand, (ii) pass the fastening cable around the forearm and lock the hooks, (iii) adjust the placement of the exoskeleton along the forearm by aligning the biological joint and mechanical axis, (iv) press on both ratchet wheels and adjust tension by turning them clockwise to secure the *eWrist* around the forearm and hand. To release the cable tension and ultimately remove the exoskeleton, the wheels have to be pulled.

*Upper arm module:* The upper arm module weighs 224 g and is attached on the proximal part of the arm (Fig. 1c). In order to fulfil our one-handed donning approach, a new spider-like mechanism has been developed. It consists of four gripping fingers and a tightening wheel. When the wheel is turned, wires running through the fingers wind around the wheel axis, which closes the fingers inwards as depicted by the orange arrows in Fig. 1c. Spring slats placed at each finger joint tend to constantly open the fingers outwards and unwind the wires around the wheel axis. A ratchet with a push button prevents the wires from automatically unwinding. To be donned, the system just needs to (i) be slightly pressed against the upper arm, (ii) held in place with the hand palm and (iii) tightened using the fingers. The gripping force can be adjusted by simply turning the wheel and therefore increasing the tension in the wires. As one side of the wires is attached to a spring, each finger can still extend outwards and thus remains compliant to changes in body shape (e.g. during biceps contraction). To release the mechanism and the tension in the wires, the ratchet needs to be disengaged by pushing a button, which will unwind the wheel.

#### Actuation

A DC motor (Maxon EC 16, $\varnothing $ 16 mm, brushless, 30 Watt) with a reduction ratio of 19:1 drives a worm drive. The motor with incorporated gearhead weights 63 g and is placed along the forearm which minimizes impediment (Fig. 1b). Mechanical backdrivability is not ensured because of the high reduction ratio, nevertheless, partial-transparency is rendered through active control. The worm drive is composed of the worm screw (in steel) and the worm wheel (in bronze), and exhibit a low friction coefficient and a high strength. The ratio of the worm drive is 25:1 leading to a total reduction ratio between the motor and the wrist axis of 475:1 (19x25). This reduction combined with the nominal torque of the motor (7.85 mNm) gives a continuous torque output at the handle up to 3.7 Nm. Moreover, the backlash between the worm screw and worm wheel can be reduced by slightly adjusting their relative position thanks to oblong fixations on the motor support. It also acts as a fail-safe in case of a high torque applied on the wrist joint and ultimately on the worm drive. In such a case, the worm screw would simply shift up and jump gears.

#### Sensors and electronics

Wrist joint velocity is computed by the motor drive, which is connected to a Hall sensor integrated within the motor together with a 128 CPT (count per turn) magneto-resistive encoder (tachometer) on the motor shaft. The current drawn by the motor is monitored by the motor drive and can be used for torque estimation and power analysis. Absolute wrist position is given by a durable Hall sensor (rotational life: up to 50M cycles) placed directly on the wrist rotational axis (Fig. 1b) with an angular position resolution of 0.058^∘^. Wrist joint torque is measured with a load cell mounted between the worm drive and the handle (Fig. 1b). The load cell is rated for a maximum force up to 50 N and has been calibrated with forces up to 30 N in both directions (extension and flexion) with a resolution of 0.0073 N. The Myo armband (Thalmic Labs) is used to record sEMG signals, which can be used in parallel with the admittance controller to trigger proportional mechanical support similar to [75, 76]. Finally, an IMU (MPU 6050) is located on the forearm module to evaluate the spatial orientation of the *eWrist*, which is required to adapt the mechanical support if the user is moving [77]. The processing and use of sEMG signals and IMU data within the controller are not discussed in the current study, which focuses on the characterization of the *eWrist*. However, the wearability evaluation included the Myo armband together with the *eWrist*.

The electronics consists of two custom-made shields, namely, the upper arm shield and the forearm shield. The upper arm shield includes the real-time micro-controller (Teensy) and a micro-computer (Raspberry Pi Zero or RPi0). The Teensy collects: force signals, absolute wrist angle, angular velocity, current consumption, battery voltage and IMU data, and runs the motor control by sending speed (or current) commands to the motor drive. The RPi0 collects sEMG data and serves as a general purpose unit to select different control algorithms or store recorded data. The forearm shield incorporates the motor drive and the IMU. The motor drive is placed close to the motor to limit the creation of electromagnetic interference (EMI) due to high commutating currents.

**Fig. 2:**
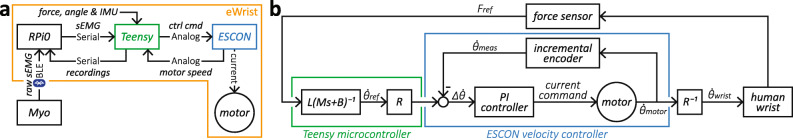
Block diagrams. **a** System architecture. **b** Admittance controller with inner velocity control loop running at 5.36 kHz on the motor drive (ESCON). The Teensy computes the reference angular velocity $\dot {\theta }_{ref}$ according to the measured force *F*_*ref*_ applied by the user on the handle and transmits it to the motor drive. The motor drive records the angular velocity $\dot {\theta }_{meas}$ of the motor shaft, combines it with the reference angular velocity $\dot {\theta }_{ref}$ reflected at the motor shaft and computes the current command for the motor thanks to an integrated PI (proportional-integral) controller. *R* is the reduction ratio of the gear stage (i.e. 475:1)

Motor and electronics are powered from a 11.1 V, 1000 mAh (11.1 Wh) lithium-ion polymer battery. The complete system architecture is depicted in Fig. 2a.

#### Control

Since the *eWrist* is not backdrivable and force is measured at the wrist joint, admittance control is a logical, simple and commonly applied controller for real-time control [78, 79]. It receives a force input and outputs a motion in response. With admittance control (Eq. 1), the dynamic behavior of the exoskeleton can be tuned with two parameters, namely virtual inertia *M* [*Nm*·*s*^2^/*rad*] and virtual damping *B* [*Nm*·*s*/*rad*]. Eq. 1 expresses the equation of motion in the time domain and its conversion to the Laplace domain with respect to angular velocity.

1 $$ M\ddot{\theta}+B\dot{\theta} = F \cdot L \quad \overset{\mathcal{L({\cdot})}}\Longrightarrow \quad \omega = \frac{L}{Ms+B} \cdot F  $$

where $\ddot {\theta }$ and $\dot {\theta }$ are the angular acceleration and angular velocity of the wrist in the time domain, respectively, *ω* the angular velocity in the Laplace domain, *L* the distance between the mechanical axis and the average pressure point of the hand on the handle (set at 8 cm), and *F* the force applied on the handle.

A discretized version of the admittance controller (Eq. 2) is implemented in the Teensy micro-controller with the Tustin transformation, which is known to preserve stability [80].

2 $$ \dot{\theta}_{ref,n} = \frac{T_{s} \cdot L \cdot \left(F_{ref,n}+F_{ref,n-1}\right)}{2M+BT_{s}}\\ + \frac{\left(2M-BT_{s}\right) \cdot \dot{\theta}_{ref,n-1}}{2M+{BT}_{s}}  $$

The current angular velocity $\dot {\theta }_{ref,n}$ depends on the past angular velocity $\dot {\theta }_{ref,n-1}$, and on the current and past force measurement *F*_*ref*,*n*_ and *F*_*ref*,*n*−1_, respectively. *T*_*s*_ is the sampling time interval. Both $\dot {\theta }_{ref}$ and *F*_*ref*_ are low-pass filtered in real-time with a moving average of window length N=20 (i.e. *f*_*co*_≈21.1 Hz at *f*_*s*_=1 kHz). The admittance controller depicted in Fig. 2b as a block diagram is the default controller of the *eWrist* used during human-robot interaction.

### Device characterization

Different aspects of the *eWrist* affecting its final performances as a rehabilitation device have been evaluated and are presented in the following section. All aspects but impedance rendering have been assessed without the exoskeleton being mounted on a forearm. Table 1 gives an overview of the main characteristics of the *eWrist*.

**Table 1:** Summary of the technical characteristics of the *eWrist*

**Performance metrics**	**Obtained values**
Forearm module weight [g]	238
Upper arm module weight [g]	224
Myo armband weight [g]	94
**Total weight [g]**	**556**
Forearm module dimensions^1^ [mm]	200 ×120×80^2^
Upper arm module dimensions^1^ [mm]	120 ×160^3^×125^3^
Output max. torque [Nm]	3.7
Output max. velocity [deg/s]	530^4^/520^5^
Output max. acceleration [deg/s^2^]	6’510^4^/7’570^5^
Force/torque range^6^ [N]/[Nm]	0-50/0-4
Force/torque resolution [mN]/[mNm]	7.3/0.58
Angular position resolution [deg]	0.058
Angular velocity resolution [rpm]	configurable
Restrained RoM [deg] (cf. Fig. 1d)	±77
Static friction^7^ [Nm]	<∣±0.1∣
Dynamic friction^8^ [Nm]	$0.00198\dot {\theta }\pm 0.0135$
Position control bandwidth [Hz]	1.74
PD steady-state error [deg]	<0.12
Autonomy^9^ [min]	125
Battery capacity [Wh]	11.1

^1^for a 1m83 tall user

^2^with palm support and fastening cable

^3^with module fingers fully extended

^4^in extension and in restrained RoM

^5^in flexion and in restrained RoM

^6^measurable by the load cell in both directions

^7^in restrained RoM

^8^for $\dot {\theta }$ up to 250 deg/s, *R*^2^=0.995

^9^in normal use

#### Maximum velocity and acceleration

Since the *eWrist* is not backdrivable, mechanical transparency (i.e. low interaction forces during human-*eWrist* interaction) can only be rendered through active control. To achieve optimal transparency, the handle should ideally move and accelerate as fast as a human wrist can. Therefore, maximum angular velocity and acceleration of the handle were assessed by deriving offline filtered angular velocity measurements recorded at 1 kHz during a maximum current impulse of 6 A [81]. Maximum velocities and accelerations were measured in both directions (extension and flexion). The angular acceleration estimate was calculated from the angular velocity via FDM (finite difference method or backward Euler method) described in Eq. 3.

3 $$ \ddot{\theta}_{n} = \frac{\dot{\theta}_{n}-\dot{\theta}_{n-1}}{T_{s}}, \quad n=\{1,2,3,...\}  $$

where $\dot {\theta }_{n}$ and $\dot {\theta }_{n-1}$ are the current and previous angular velocity measurements, *n* the control loop counter and *T*_*s*_ the sampling time interval of 0.001 s.

The discrete differentiation amplifies the quantization and discretization noise of the encoder reading such that $\dot {\theta }$ and $\ddot {\theta }$ were low-pass filtered offline with Butterworth filters^3^.

From the average of five executions, $\dot {\theta }$ and $\ddot {\theta }$ were assessed at 530 deg/s and 520 deg/s, and 6’510 deg/s^2^ and 7’570 deg/s^2^ in extension and flexion directions, respectively, as shown in Table 1.

#### Static and dynamic friction

Static friction is the motor torque (reflected at the wrist) required to move the handle at different starting angles. Static friction was identified by progressively increasing motor current in steps of 10 mA until an output movement (larger than the encoder noise) was detected. It was evaluated every 5^∘^ in both directions, i.e starting from 87^∘^ in flexion and going up to 93^∘^ in extension, and in the opposite direction. Similarly, dynamic friction was evaluated in both directions by recording mean current consumption at different angular velocities (at the wrist) ranging from 30 to 584 deg/s.

Over the restrained RoM (i.e. ±77^∘^), static friction remained below ±0.1 Nm. Variations in static friction could arise either from the worm drive, or the coupling between the motor and the worm screw. For dynamic friction, a linear relationship between angular velocity and torque (*y*=1.98×10^−3^*x*±1.35×10^−2^,*R*^2^=0.995) was identified for velocities up to 250 deg/s, as presented in Table 1.

#### Autonomy

The autonomy of a fully wearable exoskeleton is a significant aspect of its usability and is a common performance metric for electronic equipment. In our case, considering non-spastic stroke survivors, the autonomy was defined as the time during which the device can continuously move a passive hand in extension and flexion when it is placed horizontally (Fig. 1d) and a given battery (11.1 Wh) is used. A total electrical energy of 2’800 J (i.e. 0.78 Wh) was used to move the passive hand of a 1m83 tall user during 10 min at a constant speed of 25.7 deg/s.

To assess our on-board energy measurement and the practical battery capacity, we simulated a whole autonomy trial (i.e. from battery fully charged until fully discharged) by actuating the *eWrist* in water. To this end, the *eWrist* was equipped with a paddle fixed at the end of a lever and constantly immersed into water. The lever was directly fixed to the load cell. The lever length, the paddle surface area and the angular velocity (set at 35.4 deg/s) were adjusted to yield maximal mechanical resistance while staying within the device’s capability. Following this trial, a total electrical energy consumption of 35’060 J (i.e. 9.75 Wh) was measured, which is reasonably close to the theoretical capacity of the battery (i.e. 11.1 Wh) considering its state of use.

The practical autonomy of the *eWrist* was inferred with the aforementioned conditions to 125 min (i.e. 10*35’060/2’800) as shown in Table 1.

#### Position bandwidth

The closed-loop position bandwidth evaluates the dynamics of the system and shows how quickly the device can react to fast and small changes in direction. In this assessment, the handle was PD controlled to follow a sinusoidal trajectory with a constant amplitude of 5^∘^ and increasing frequency from 0.1 to 6 Hz. The PD controller was implemented specifically for this assessment and was tuned to render maximum dynamic performance while remaining stable under these specific conditions.

The position bandwidth was evaluated at 1.74 Hz (at -3 dB) as presented in Table 1. At that frequency, the phase shift was 61.6^∘^.

#### Steady-state error

The steady-state error evaluates how precisely the handle can be controlled to reach a given angular position. For this assessment, a PD controller was implemented and step impulses from 40^∘^ in flexion to 40^∘^ in extension, and vice-versa, were executed in an alternating manner. Once the handle stabilized, the error was determined and the process repeated over seven trials for averaging. The PD controller was tuned to reach the target position as fast as possible without overshooting.

The steady-state error was on average lower than 0.12^∘^ as shown in Table 1. We also estimated steady-state error with the same PD controller used for assessing position bandwidth (i.e. identical tuning parameters *K*_*p*_ and *K*_*d*_), which yielded an error lower than 0.31^∘^ on average.

#### Impedance rendering

The admittance controller described in Eq. 2 can be tuned with two parameters, namely virtual inertia *M*_*virt*_ and virtual damping *B*_*virt*_ to render various mechanical impedance ranging from transparent to resistant. The ability of our device to render a low and a medium impedance behaviour was assessed through impedance planes. A low impedance plane (or transparency plane) captures the lower apparent impedance boundary of the device based on measurements during human-robot interaction. It indicates visually, through its flatness, whether the device is transparent or resists the movements of the user [82, 83]. The steeper the plane, the more resistant the interaction.Impedance planes were generated with the *eWrist* worn on the forearm and while performing extension and flexion movements repetitively during 1) transparent rendering and 2) resistive rendering. In the transparent rendering, *M*_*virt*_ and *B*_*virt*_ were set as low as possible to allow a stable human-robot interaction, while for the resistive rendering they were set so that the torque applied by the experimenter would remain in an acceptable range for the *eWrist*. The ratio between *M*_*virt*_ and *B*_*virt*_ was also adjusted to optimize the stability of the human-robot interaction. Angular acceleration $\ddot {\theta }_{int}$ was calculated via FDM from the angular velocity $\dot {\theta }_{int}$. Interaction force *F*_*int*_, $\dot {\theta }_{int}$ and $\ddot {\theta }_{int}$ were low-pass filtered offline with Butterworth filters^4^. The recordings and estimates were then fitted with a multiple linear regression model presented in Eq. 4.

4 $$ F_{int} = M_{app} \cdot \ddot{\theta}_{int} + B_{app} \cdot \dot{\theta}_{int}  $$

where *M*_*app*_ and *B*_*app*_ are the apparent inertia and damping felt by the user during human-robot interaction.

**Fig. 3:**
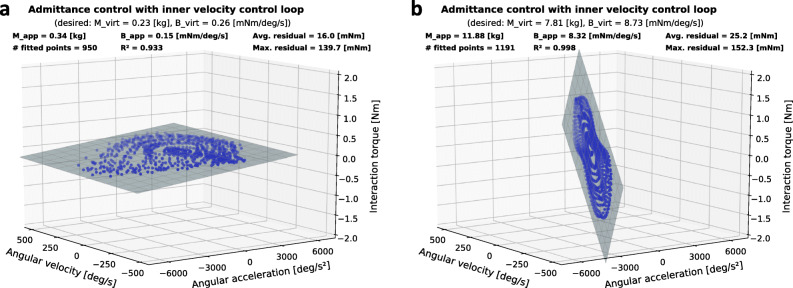
Impedance planes obtained during human-*eWrist* interaction (repetitive extension and flexion movements) for two different sets of virtual inertia *M*_*virt*_ and damping *B*_*virt*_. **a** Low mechanical resistance (i.e. high transparency) where *M*_*virt*_ and *B*_*virt*_ were set to 0.23 kg and 0.26 mNm/deg/s, respectively. **b** High mechanical resistance to movements where *M*_*virt*_ and *B*_*virt*_ were set in the admittance controller to 7.81 kg and 8.73 mNm/deg/s, respectively

The force-motion recordings $\left (F_{int}, ~\dot {\theta }_{int} ~\text {and} ~\ddot {\theta }_{int}\right)$ and the fit model are then plotted as points and as a plane, respectively, in a 3 dimensional plot (Fig. 3). To validate the assumed linearity of the impedance plane model, the residuals of the multiple linear regression must be small, i.e. the trajectory points must lie close to the fitted plane. The axes of the 3 dimensional plot are scaled up to the maximum angular velocity and acceleration found previously during maximum current impulse. If velocity/acceleration recordings were to reach the limits of the axes, the motor of the *eWrist* would have been driven into saturation [83]. In any case, the limits of the axes cannot be crossed since the actuation system is not backdrivable.

The two different dynamic behaviors of the admittance controller during human-*eWrist* interaction are shown in Fig. 3. In Fig. 3a (transparent rendering), where inertia (*M*_*virt*_=0.23 kg) and damping (*B*_*virt*_=0.26 mNm/deg/s) were set low, the flatness and large spread of the plane indicate that the user could freely (i.e. with low interaction torques up to 0.34 Nm) and rapidly (i.e. with high angular velocities and accelerations up to 456 deg/s and 7016 deg/s^2^, respectively) execute movements while wearing the device. Whereas in Fig. 3b (resistive rendering), the user experienced a rather large inertia (*M*_*virt*_=7.81 kg) with high damping (*B*_*virt*_=8.73 mNm/deg/s) when performing extension and flexion movements. Therefore, a steep plane with high interaction torques (up to 1.59 Nm) and low velocities (up to 157 deg/s) can be observed.

In both conditions, the apparent inertia *M*_*app*_ (0.34 kg and 11.88 kg) felt by the user are about 50% larger than the virtual inertia *M*_*virt*_ (0.23 kg and 7.81 kg) set in the controller. Interestingly, the apparent damping *B*_*app*_ (0.15 mNm/deg/s and 8.32 mNm/deg/s) remained lower than the virtual damping *B*_*virt*_ (0.26 mNm/deg/s and 8.73 mNm/deg/s) in both conditions. Moreover, in both conditions, low residuals (16.0 mNm and 25.2 mNm) and high R^2^ (0.933 and 0.998) indicate that the human-*eWrist* interaction remained linear over the whole RoM.

### Functionality and wearability testing

The independent donning and doffing of the *eWrist* was tested via a wearability evaluation in healthy participants and stroke survivors by means of needed time to execute the tasks, and questionnaires.

#### Subjects

Fifteen healthy subjects (7 females and 8 males, mean age: 26 ±3.4, ranging: [22, 33] years) and two stroke survivors S1 and S2 were recruited (both males, age: 68 and 52 years, FM-UE: 44 and 41, both left-arm impaired and both suffered a haemorrhagic stroke 167 and 113 months ago, respectively). In the healthy participants, eight were identified as right-handed, five as left-handed and four as ambidextrous according to the Edinburgh inventory [84]. Both stroke survivors were identified as right-handed. The study was approved by the institutional ethics committee of the ETH Zürich. All subjects gave signed, written informed consent in accordance with the Declaration of Helsinki before participating in the experiment.

#### Experimental protocol

The experiment consisted of donning and doffing the *eWrist* exoskeleton, independently, with a single hand, on the right arm for the healthy participants, and on the left arm for the two stroke survivors. The donning procedure consisted of placing: 1) the Myo armband, 2) the forearm module and 3) the upper arm module. During the experiment, the participants were asked to speak out their thoughts aloud, i.e. to explain what they were doing while they were doing it. This so called *“Think Aloud Method”* [85, 86], encourages the verbalisation of mental processes and enhances the feedback collection by the two experimenters who were present during the whole session. To familiarize the participants with the *Think Aloud Method*, they were asked beforehand to take the dimensions of their forearm and upper arm with a ruler, while explaining what they were doing. They were then introduced to the purpose and working principle of the *eWrist*, and donning and doffing were demonstrated. They were given two trials to fully don and doff the *eWrist*. Then the time needed to don and doff the *eWrist* was recorded for two subsequent trials marked as 1 ^*st*^ and 2 ^*nd*^ trial in Table 2. For both the donning and doffing, time was started once the participant touched the device (*eWrist* or Myo armband) and stopped when he/she released it. During the trials, participants were asked not to rush, but simply to execute the task at normal speed. Finally, they had to fill in questionnaires assessing the donning/doffing usability of the device.

**Table 2:** Donning and doffing time

**Participant**	**Donning**		**Doffing**	
	1 ^*st*^ trial	2 ^*nd*^ trial	1 ^*st*^ trial	2 ^*nd*^ trial
Healthy	79.3 ±25.9	61.5 ±15.1	27.7 ±7.0	24.0 ±6.2
S1 (FM: 44)	54	54	22	22
S2 (FM: 41)	127	113	31	32

The average time, in seconds and per trial, the healthy participants required to don and doff the *eWrist*, and the individual time of the two stroke survivors S1 and S2.

#### Questionnaires

Three different questionnaires were completed by the participants just after the test to quantify their subjective opinion on the donning/doffing procedure.

The first questionnaire is a standard System Usability Scale (SUS), which is a quick, simple and reliable tool for measuring usability [87, 88].

The second questionnaire (called SUS customized) has been customized for our device evaluation and is based on the same scoring scheme as the SUS but incorporates 32 questions instead of 10. The questions were orientated around five different aspects of usability, which are typically assessed in such evaluations [89, 90], namely: learnability, efficiency, memorability, errors and satisfaction. Each of these aspects were evaluated independently.The last questionnaire is the Raw NASA-Task Load Index (RTLX) [91], which is a six-dimensional scale designed to assess the workload experienced during a task with the following aspects: mental demand, physical demand, temporal demand, performance, effort, and frustration [91, 92]. In its full version (i.e. not raw), the NASA-Task Load Index (TLX) incorporates a weighting procedure of these 6 aspects, however, for the sake of simplicity, we omitted this procedure and weighted all aspects equally. The RTLX has been shown to be highly correlated with the TLX [93, 94]. In our analysis, each aspect was considered individually.

Moreover, all participants could leave written comments at the end of the questionnaires.

## Results

A positive and promising outcome from the wearability evaluation is that all participants (healthy and stroke) were able to don and doff the device independently, with a single hand, and after only two practice trials.

### Donning/doffing time

Table 2 summarizes the average time healthy participants needed to don and doff the *eWrist* during their 1 ^*st*^ and 2 ^*nd*^ trial, and the individual time performance of the two stroke survivors S1 and S2. A significant time improvement can be observed between the two subsequent trial in both donning (paired t-test: p <0.001) and doffing (paired t-test: p <0.01) with healthy participants. With stroke survivors, participant S1 was remarkably fast and consistent over the two trials, performing better than most of the healthy subjects. On the other hand, S2 was much slower but improved his time performance during the donning over the two trials.

No significant time difference could be observed between right-handed, left-handed and ambidextrous participants both in donning and doffing.

### Questionnaires

**Fig. 4:**
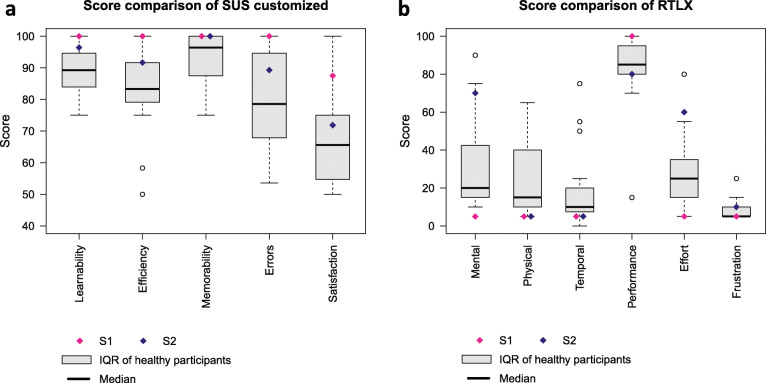
Scores comparison derived from questionnaires for all participants (healthy and stroke). **a** Scores from the customized SUS questionnaire. The average score over all aspects is 83.1 ±8.2. **b** Scores from the RTLX questionnaire. The average workload score excluding *Performance* [108] is 22.3 ±9.5

The average score of the SUS questionnaire is 82.0 ±7.1 for healthy participants, and 97.5 for S1 and 80 for S2, with a score over 68 being considered above average [95].In Fig. 4a are shown the scores of each aspect of the customized SUS questionnaire for both healthy and stroke subjects. Generally, all subjects found it simple to learn how to execute the tasks (*Learnability* score: 90.6 ±7.7), i.e. how to correctly place and tighten/untighten the forearm and upper arm fixations. They also found the fixation systems efficient and secure (*Efficiency* score: 84.8 ±13.9), and they could easily remember how to perform the overall donning/doffing procedure (*Memorability* score: 92.7 ±8.7). On the other hand, some participants found they were likely to make errors when donning the device (*Errors* score: 81.9 ±14.8), and were not fully satisfied with the exoskeleton (*Satisfaction* score: 68.6 ±13.9) because it was hindering their forearm/hand movements. It was also received low scores for aesthetics, physical proportions and weight.

Figure 4b depicts the scores of the RTLX questionnaire issued to the participants right after the trials. The score ranges from 0 to 100 and reflects the workload of each aspect. A low score indicates that perceived workload was low. A high score in *Performance* means that the participants found they were successful in accomplishing the task. Mental and physical workload (score: 32.6 ±26.1 and 24.1 ±19.9, respectively) exhibit a large variability for both healthy and stroke participants where the mental demand was much higher for S2 (score: 70) than for S1 (score: 5). In the same vein, the effort required to execute the task was perceived much differently by S2 (score: 60) than S1 (score: 5). Other than that, all participants found they could accomplish the task rapidly without being rushed (*Temporal* score: 18.2 ±21.4), they found they were successful (*Performance* score: 84.7 ±20.2) and not frustrated during the donning and doffing (*Frustration* score: 8.2 ±5.3).

### Subjective feedback

Below are subjective feedback and observations collected during the donning/doffing which are to be considered for future prototype versions:

- During their first trials, participants were struggling to appropriately place the forearm module so that both the mechanical axis and biological joint are aligned.
- The donning phase of the forearm module where the hooks must be locked was sometimes problematic since the device must be balanced on the forearm while it tends to fall towards the side of the actuator.
- Some discomfort appeared (tightening cable lacerating the skin, blood vessels blocked, skin squeezed) because of size mismatch. Also, for users with small hands, turning the rather large wheel of the tightening system on the upper arm module was difficult.
- The forearm fixation tends to generate stress on the skin during pronation and supination movements.
- Some female users found it tedious to turn and pull the ratchet wheels on forearm and handle fixations.
- The stroke survivors experienced slight difficulties to pass their fingers through the loop formed by the palm support if the latter was not completely loose (cf. Fig. 1b).

In the comments left by the participants, positive feedback were given regarding the ease of use and comfort of the device. On the other hand, some commented size mismatches, excessive perceived weight (especially because they were holding their arm above the table), movement constraints and a need for design improvement especially in terms of aesthetics.

## Discussion

In this paper we have presented the development, characterization and wearability evaluation of a fully portable, powered one DoF wrist exoskeleton designed for independent and unsupervised training. The results of the characterization showed that the current prototype fulfils the technical requirements of output torque (up to 3.7 Nm), angular velocity (up to 530 deg/s) and RoM (154^∘^ or up to 215^∘^ if required), distal weight (238 g for forearm module) and autonomy (125 min) as previously specified in the literature. Furthermore, the wearability evaluation revealed that all participants (healthy and stroke) embraced the device and were able to don and doff it independently and quickly after a few practice trials.

### Design choices and performance characterization

Our approach of directly integrating the actuator and its drive locally at the wrist has the advantage of a rather simple implementation and good control of the wrist joint (PD steady-state error <0.12^∘^). However, the motor alone (69 g) accounts for about 29% of the forearm module weight (238 g) and is therefore a major contributor of the weight placed distally on the arm. Fixing the second module on the upper arm reduces the weight distally and facilitates donning with the other hand, but still impacts arm motion in patients. This could be avoided by moving it to the back or less affected body side [72, 96, 97], however, the further the exoskeleton is removed from accompanying modules, the more difficulties arise for donning and doffing independently. Thus, our solution is a compromise between good usability for donning/doffing and reducing the weight attached distally to the affected arm. The weight of the forearm module is comparable or lower than for other similar devices [32, 98, 99].

The dynamics assessment has demonstrated that the angular velocities and accelerations achievable with the *eWrist* in the restrained RoM are comparable to those observed in healthy skilled workers which perform typical manual activities [100]. High achievable velocities and accelerations are necessary to render transparency. Despite a rather low position bandwidth of 1.7 Hz our impedance planes show that the implemented admittance controller can stably (cf. high R^2^ and low residuals) provide transparent or resistive dynamic behaviour, which is important for accommodating different rehabilitation training settings [101]. The capacity to provide all of these training modalities is important for haptic rehabilitation devices [102, 103] for (i) training a wide range of impairments (i.e. from plegic to moderately impaired function), and (ii) quantitatively assessing the patient’s ability to perform movements without being disturbed by the device dynamics [82].

PD controllers were implemented in both steady-state error and position bandwidth assessments and were tuned for maximum performance in each case. Although proportional *K*_*p*_ and derivative *K*_*d*_ tuning parameters were set to different values for each assessment, their ratio was kept the same to preserve stability (*K*_*p*_/ *K*_*d*_=10 in both cases). Moreover, *K*_*p*_ and *K*_*d*_ were 45% larger for the position bandwidth assessment compared to the steady-state error assessment in order to exhibit a more dynamic behavior. The two PD controllers were implemented for the sole purpose of performing these assessments and tuned independently to demonstrate the best capability of the device in each experimental context. Only the admittance controller is used during human-robot interaction, and dictates the experience of the user with the device.

Our autonomy assessment is comparable to other studies [32, 39, 53] and would provide an extensive training dose for the user. However, the obtained autonomy must be interpreted with caution because it depends on the movement regime, for example, higher interaction velocities or higher interaction forces might arise if the hand is not completely passive. Surprisingly, we observed during the autonomy assessment that for a given time and with a substantially larger angular velocity (+42%), the energy consumption was reduced (-5%), revealing a non-linear effect which decreases with increasing velocity. This observation could partially explain the large disparity (-42%) between the desired virtual damping *B*_*virt*_ and the measured apparent damping *B*_*app*_ seen in Fig. 3a but not in Fig. 3b. With larger velocities this non-linear effect is lower, leading to a lower apparent damping felt by the user.

In the same vein, the large discrepancies (about +50%) observed in both renderings (i.e. transparent and resistive) between the virtual inertia *M*_*virt*_ and the apparent inertia *M*_*app*_ can be mainly explained by the intrinsic mechanical feature of our design, which requires that an interaction force needs to be applied first in order to illicit a motion. In the time delay (due to processing) between the force measurement and the handle motion, the force increases. And stronger forces will cause stronger friction between the gears and eventually resistance to the movement, thus leading to a larger apparent inertia experienced by the user compared to the one initially set in the controller. The steel-bronze combination for the worm drive is a fair compromise between low friction coefficient and high strength [104]. Nevertheless, special attention must be given to optimising the manufacturing of these parts to keep their weight low. Moreover, the first-order characteristics of Eq. 1 also introduces a time lag in the control command which is directly linked to the inertia term. It would thus be tempting to minimize or even suppress this term to decrease time lag, however, we observed empirically that both terms (inertia and damping) are required to stabilize the exoskeleton during human-robot interaction. More specifically, stability was enhanced when the ratio between inertia and damping remained constant, as shown in other studies [105–107]. Finally, since the worm screw can shift up relative to the worm wheel due to the oblong fixation points, our experience showed that the safety of the user’s wrist and the mechanics are preserved in case of unexpected high torque.

### Wearability evaluation and general considerations

The effort directed towards the development of adjustable attachment systems which ease the donning and doffing procedure of the *eWrist* was positively received by the participants according to the scores obtained in our questionnaires. Although not standardized, the customized SUS questionnaire allowed us to get a better understanding of which specific aspects were favored and which were disliked. Encouragingly, the majority of participants quickly endorsed the mechanisms and found them efficient in terms of gripping force and adjustability. Generally, the doffing was found more straightforward than the donning. Stroke survivors judged wearability similar to healthy participants in the customized questionnaire. However, one clear limitation of our study is that we tested only two patients with moderate to minor impairment. In order to generalize our results to stroke patients, it would be valuable to also test wearability in more severely impaired patients. One important difference between the two cohorts was that healthy participants, but not stroke survivors, found their movements to be hindered by the device, most likely reflecting a difference in the perceived benefit of motor assistance via the exoskeleton.

As mentioned in the design review and also clearly expressed in the feedback, a critical phase during the donning is the correct placement of the forearm module to match the biological joint and mechanical axis of the *eWrist*. Most of the participants struggled with this aspect during the first four trials. During this phase, the forearm module must be balanced on the forearm and the hooks of the attachment system locked. However, the combined weight of the actuator, the gear drive and the load cell, all located on the same side of the module, tends to tip the device over. Nonetheless, our experience suggests that with slightly more practice both of these phases can easily be mastered.

According to a survey of 22 studies scoring mechanical tasks with the TLX [108], the obtained score of 22.3 in the RTLX questionnaire (average workload score without considering *Performance*) is below the 25th percentile of the scores (i.e. better than 75% of all scores). Nevertheless, despite this encouraging result, the scores comparison in Fig. 4b reveals that stroke survivors perceived mental and physical demands of donning/doffing much differently from healthy participants. This disparity, and more generally the wearability evaluation, should be further assessed by testing the device with more stroke survivors of different impairment levels and over several sessions. Nonetheless, it has been shown that the most critical usability problems are likely to be detected in the first few subjects, and that the likelihood of uncovering new problems decreases as more and more subjects participate [109]. In our usability study, we consistently observed that difficulties encountered by healthy participants affected stroke subjects in a similar manner.

The weight of the exoskeleton was found to be acceptable. The rating was sometimes biased when participants would hold their whole arm over the table during the donning instead of laying it down, thus increasing their weight perception. Unfortunately, some participants felt discomfort mainly due to size mismatch. This can be addressed by tailoring the device to the individual user. For this study, two *eWrist* of different dimensions were built, one for the right arm and one for the left. Based on anthropometric measurements (width, length and circumference) of the forearm, the wrist and the hand, an individualized exoskeleton can be printed. Tailor-made manufacturing with 3D printing techniques has already been adopted in community settings to offer simple prosthetics for impaired children [110] and could potentially be applied for powered and more complex robots [111]. Nevertheless, although the structure and 3D printed parts can be adapted, the electronics, load cell and actuator remain the same and would not properly suit small patients (i.e. <1m60 tall).

There were a number of general limitations to the wearability assessment. First, introducing the concept of the device before its assessment might have biased the participants towards higher ratings regarding functionality. Second, certain participants might have evaluated their own performance rather than the actual wearability of the device. Third, the wearability assessment was also limited in its design since participants were only evaluating the device during a single session. For instance, it would have been worthwhile to evaluate whether participants had memorized the procedure by retesting them after a week. Finally, the use of the *Think Aloud Method* conjointly with the observations of the two experimenters allowed identification of where participants were experimenting difficulties in the task. However, even though participants were given preparation in verbalizing their thoughts, the use of this method with naive users had a tendency to slow down the execution time, especially with S2. Additionally, one has to keep in mind that we only evaluated the donning and doffing of the device but did not yet test its usability within a rehabilitation setting. Even though wrist extension/flexion function is highly relevant for post stroke recovery [49], only supporting this movement in such a setting might limit some activities.

In its current form, the *eWrist* is an important preliminary step towards a rehabilitation technology that could be donned, used and doffed independently by the patient in unsupervised settings, and which would complement a conventional therapy. Target patients would ideally start training with this device in the acute or sub-acute phase post stroke. The main inclusion criterion is low spasticity (i.e. MAS <3). However, patients who will likely benefit the most are those that have some remaining EMG activity in the forearm muscles and suffer from impaired hand and/or wrist function. In the initial phase, patients would use the device in a supervised manner, but as rehabilitation progresses and their impairment decreases, they would use the device more independently in daily life settings. As currently envisioned, rehabilitation training with the *eWrist* will be in the form of a visuomotor task where the wrist angle of the exoskeleton is visualized as a cursor on a computer display and the patient performs wrist extension and flexion movements to move the cursor to different targets [112], with an adaptive level of mechanical support from the exoskeleton based on sEMG amplitude. The control of robotic devices with sEMG signals have been extensively studied and one of the most preferred approach is to proportionally match sEMG to position [75] or force [34, 113]. We believe that a visual feedback combined with the mechanical support can not only reinforce sensorimotor loops and enhance the recovery process, but perhaps more importantly, boost motivation. Moreover, the wearable aspect of the device gives more freedom to the user and could easily be combined with a smartphone or a tablet.

## Conclusion

In the context of a robotic-directed therapy in unsupervised settings, donning a medical device is the very first barrier a patient will have to face if he/she were to train independently. Therefore, it is essential that this first step is straightforward and keeps the user’s motivation high. In this paper, we have demonstrated that the performance of our device is similar or better than other fully wearable exoskeletons for wrist training, but more specifically, we have drawn attention to the problem of the independent donning/doffing of an upper limb exoskeleton and have brought new insights on possible user-friendly and innovative mechanisms which ease this procedure.

## Data Availability

The datasets used and/or analyzed during the current study are available from the corresponding author on reasonable request.

## Abbreviations

ADL: Activities of daily living
DoF: Degree of freedom
RoM: Range of motion
sEMG: surface electromyography
IMU: Inertial measurement unit
CPT: Counts per turn
RPM: Rotation per minute
EMI: Electromagnetic interference
PI: Proportional-integral
FDM: Finite difference method
FM-UE: Fugl-Meyer for upper extremities
MAS: Modified Ashworth Scale
SUS: System usability scale
NASA-TLX: NASA-task load index.

## Footnotes

1 Polylactic acid

2 Thermoplastic polyurethane

3 $\dot {\theta }$ and $\ddot {\theta }$ with a 2 ^*n**d*^ order 10 Hz and 20 Hz cut-off frequency, respectively.

4
*F*_*int*_, $\dot {\theta }_{int}$ and $\ddot {\theta }_{int}$ with a 2 ^*n**d*^ order 5 Hz, 10 Hz and 20 Hz cut-off frequency, respectively.
